# Association between *MTHFR* gene *1298A>C* polymorphism and breast cancer susceptibility: a meta-analysis based on 38 case-control studies with 40,985 subjects

**DOI:** 10.1186/s12957-016-0978-2

**Published:** 2016-08-27

**Authors:** Jinghong Zhang, Lijun Zhang, Guangming Li

**Affiliations:** Department of General Surgery, Beijing TongRen Hospital, No.1 Jia Dong Jiao Min Xiang, Dongcheng District, Beijing, 100730 China

**Keywords:** *MTHFR* gene *1298A>C* polymorphism, Breast cancer, Gene polymorphism, Meta-analysis, One-carbon metabolism, Variant

## Abstract

**Background:**

Studies investigating the association between the *methylenetetrahydrofolate reductase* (*MTHFR*) gene *1298A>C* polymorphism and the risk of breast cancer have reported inconsistent results. So, we performed this updated meta-analysis and tried to give a more precise estimation of association between *MTHFR* gene *1298A>C* polymorphism and breast cancer susceptibility.

**Methods:**

Relevant studies published before 1 January 2016 were identified by searching PubMed and EMBASE. The strength of relationship between the *MTHFR* gene *1298A>C* polymorphism and breast cancer susceptibility was assessed using odds ratio (OR) and corresponding 95 % confidence interval (95 % CI). The meta-analysis was performed using Stata 11.0 software.

**Results:**

A total number of 38 case-control studies including 18,686 cases and 22,299 controls were identified. No association was found in five genetic models (dominant model: OR = 0.99, 95 % CI 0.99–1.00, *P* = 0.218; recessive model: OR = 1.00, 95 % CI 0.97–1.02, *P* = 0.880; homozygote genetic model: OR = 0.99, 95 % CI 0.98–1.01, *P* = 0.390; heterozygote genetic model: OR = 0.99, 95 % CI 0.97–1.00, *P* = 0.138; and allele contrast genetic model: OR = 0.99, 95 % CI 0.98–1.01) for *MTHFR* gene *1298 A>C* polymorphism and breast cancer susceptibility. In the subgroup analysis stratified by source of control, decreased risk of breast cancer was found in studies with hospital-based controls in dominant model (OR = 0.98, 95 % CI 0.96–1.00, *P* = 0.037).

**Conclusions:**

Our meta-analysis suggested that there is no significant association between *MTHFR* gene *1298A>C* polymorphism and breast cancer susceptibility for overall population.

## Background

Breast cancer is the most frequently diagnosed cancer among women, which contributed to 25 % of all cancer cases in women worldwide, and it is the leading cause of female cancer-related death [1]. In UK, 48,034 women were diagnosed as breast cancer holders in 2008, and in USA, more than 2.8 million women suffered from breast cancer in 2015 [2, 3]. In China, breast cancer mortality have also raised quickly in recent years, from 3.53/100,000 in 1990–1992 to 4.25 in 2012 [4]. The high morbidity and mortality of the disease lead to increasing global public health burden gradually. It is widely accepted that several factors, such as hormonal, environmental, and genetic factors as well as their interactions contribute to the onset of breast cancer [5, 6]. In 1993, mutations in breast cancer (*BRCA1)* gene were suggested to be linked with high incidence of breast cancer in some families [7]. Since then, many susceptible genes involved in initiation and evolution of breast cancer have been researched, and one of them, the *methylenetetrahydrofolate reductase* (*MTHFR*) gene has been widely studied.

The *MTHFR* locus locates on chromosome 1 at the end of short arm (1p36.6), which encodes enzymes relevant to folates metabolism. The enzyme encoded by *MTHFR* gene takes part in the irreversible conversion of 5,10-metylenetetrahydrofolate to 5-methyltetrahydrofolate, which plays a crucial role in homocysteine remethylation to methionine [8]. Previous studies have indicated that functional single nucleotide polymorphisms (SNPs) of *MTHFR* gene participate in the folate-metabolizing genetic pathway and are fundamental during the synthesis, repair, and methylation process of DNA, RNA, and protein, which may affect folate and vitamin B_12_ level [9, 10]. Of these SNPs, *1298A>C* polymorphism is caused by A to C transition in exon 7 and results in alanine in substitution of glutamine at codon 429 of the protein [11]. Subjects with mutated *MTHFR 1298A>C* genetic polymorphisms have higher plasma level of homocysteine [12] and may be more susceptible to different kinds of cancers, including breast cancer.

Many studies have investigated the association between *MTHFR* gene *1298A>C* polymorphism and breast cancer risk. However, the results are inconsistent, with some studies found significant association [13, 14], while others were not [15, 16]. Although previous meta-analysis has tried to clarify the association [17], recently, several new case-control studies have been published [18–20]. In order to avoid the limitations of single case-control studies and provide renewed evidence, we performed this updated meta-analysis and tried to give a more precise and comprehensive estimation of association between *MTHFR* gene *1298A>C* polymorphism and breast cancer susceptibility.

## Methods

### Data sources

Two databases were electronically searched, including PubMed and EMBASE, to retrieve studies analyzing the association between breast cancer susceptibility and *MTHFR* gene *1298A>C* polymorphism until January 1, 2016. Searching terms were “breast cancer” or “breast neoplasm”, in combination with “*methylenetetrahydrofolate reductase*” or “*MTHFR*” or “*MTHFR A1298C*” or “*MTHFR 1298A>C*” or “*rs1801131*” or “*Glu429Ala*”, and in combination with, “polymorphism” or “variant” or “genotype” or “allele”. We also hand-checked the reference lists of all the included studies to make sure no study was missed. Two researches conducted the searches independently. If several publications carried out among same patients and controls, we only included one study with the most complete data.

### Inclusion criteria

We first performed initial screening of titles and abstract. A second round screening was based on full-text reviews. Studies were considered eligible if they met the following criteria: (1) it was a case-control study in design; (2) it evaluated the *MTHFR* gene *1298 A>*C polymorphism and breast cancer susceptibility; (3) breast cancer was pathologically confirmed for all of the patients; (4) sample sizes and individual genotype frequencies in cases and controls were available; and (5) cases and controls should be matched.

### Exclusion criteria

Researches were excluded if they met any one of the following criteria: (1) data came from reviews or abstracts; (2) genotype and allele frequencies were both unavailable; (3) subjects with other malignant tumor were included in controls; (4) repeatedly published literature; (5) not breast cancer susceptibility outcome; and (6) controls were chosen from women with a family history of breast cancer or with other kinds of malignant tumors.

### Data extraction and quality assessment

Two reviewers independently searched and selected literature, and then, extracted relevant data according to a data extraction form. Disagreements were solved by discussion until consensus was made. The extracted data including the first author, year of publication, country of origin, ethnicity of the study population, source of control, sample size, the genotype and allele frequencies of the *MTHFR* gene *1298A>C* polymorphism, and information of Hardy-Weinberg equilibrium (HWE) in control groups. Different ethnicity descents were categorized as Caucasian, Asian, African, and if studies were with more than one ethnicity, they were categorized as mixed ethnicity.

For each included study, the quality assessment was conducted according to the STrengthening the REporting of Genetic Association (STREGA) studies). If the study met all or most of the criteria in this approach, it would be classified as “++” or “high quality”. For study in which some of the criteria were fulfilled and the others were not likely to change the results and conclusions, it would be graded as “+” or “moderate quality”. For studies fulfilled few or no criteria and the results were thought to be with non-ignorable bias, it would be classified as “−” or “low quality” [21].

### Statistical analysis

Data analysis was conducted using STATA 11.0 software (Stata Statistical software, College Station, TX, USA, www.stata.com). Odds ratio (OR) and its corresponding 95 % confidence intervals (95 % CI) were used to evaluate the strength of association between *MTHFR* gene *1298A>C* polymorphism and breast cancer susceptibility. Heterogeneity among included studies was tested using chi-square-based *Q* test and *I*^2^ test. *P*_het_ < 0.05 or *I*^2^ > 50 % were considered as statistically significant for heterogeneity. The Mantel-Haenszel method was used for fix-effect model if no heterogeneity was found. Otherwise, the DerSimonian-Laird random-effect model was used. Fix-effect model considers that across all studies, the genetic factors have similar effects on genetic disorder susceptibility and the observed differences among studies are caused just by chance [22]. Random-effect model considers that different studies may have substantial diversity, and it calculates within- as well as between-study difference [23]. Five comparison genetic models were used to assess the association between *MTHFR* gene *1298A>C* polymorphism and breast cancer susceptibility. We assessed the dominant model (AA + AC vs. CC), recessive model (AA vs. AC + CC), allele contrast genetic model (A vs. C), the heterozygote comparison (AC vs. CC), and the homozygote comparison (AA vs. CC). *P* < 0.05 showed the statistical significance. HWE was tested for included studies if no relevant information was provided in original research. Sensitivity analyses were conducted by omitting individual studies sequentially. Moreover, we performed subgroup analysis stratified by ethnicity, source of control, and deviation from HWE. Publication bias was quantitatively assessed by Egger’s linear regression test [24] and visual inspection of Begg’s funnel plots.

## Results

### Literature search

**Fig. 1:**
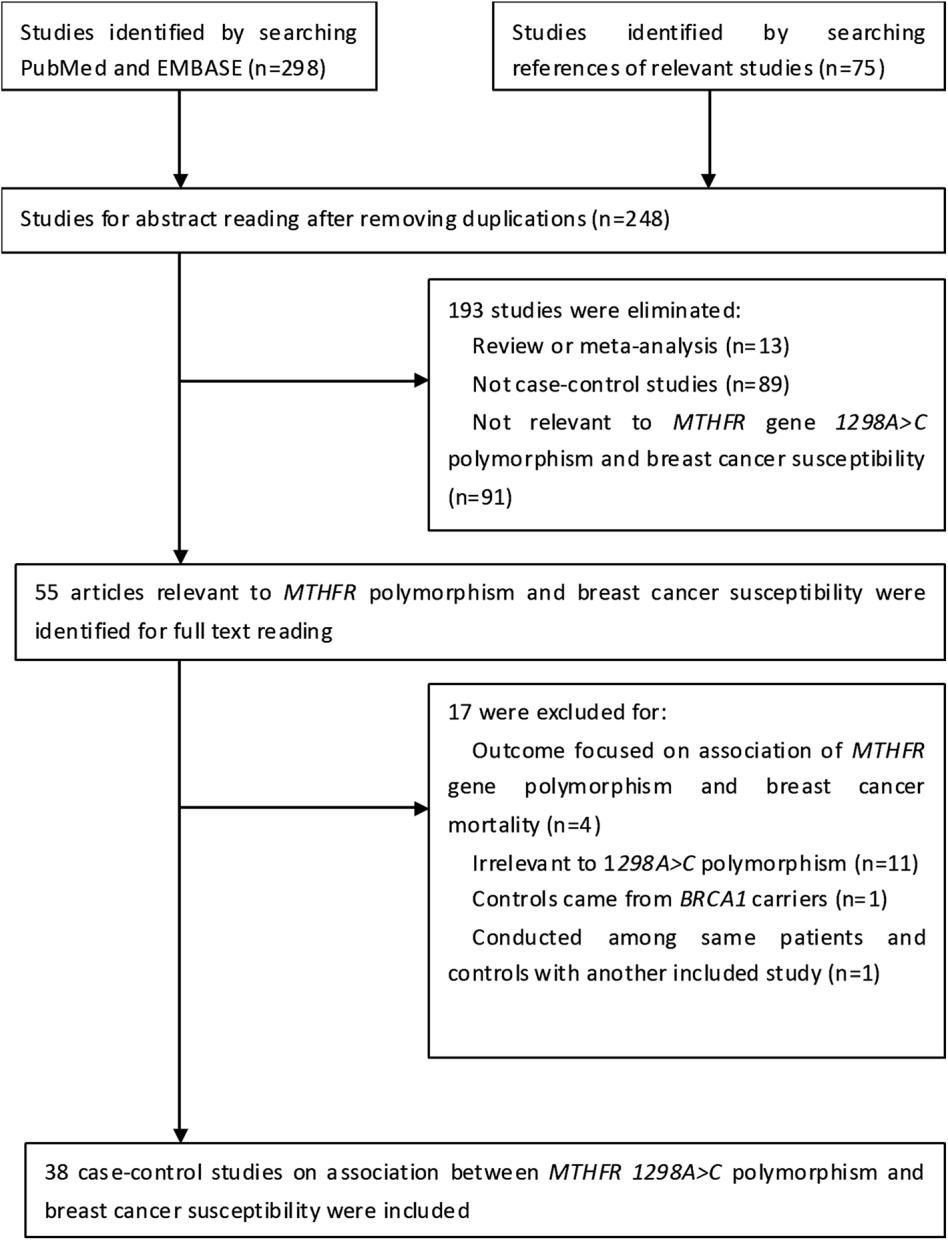
Flow chart of data selection

We initially identified 373 potentially relevant studies from searching the two databases and the reference lists of relevant studies. Firstly, we eliminated duplications, and after this procedure, 248 studies were retained. After reading the titles and abstracts, we excluded 193 studies. Among them, 89 were not case-control studies, 91 were irrelevant to *MTHFR* polymorphism or breast cancer susceptibility, and 13 were reviews or meta-analysis. Then, we read the full texts of the 55 retained articles and 17 were excluded. Of them, 11 was irrelevant to *1298A>C* polymorphism, four focused on breast cancer mortality, one conducted among the same patients and controls with another study, but provided less completed data, and for one study, the controls were chosen from *BRCA1* carriers. We finally identified 38 case-control studies eligible for the meta-analysis [13–16, 18–20, 25–55], including 18,686 cases and 22,299 controls. A flow chart of data selection was presented in Fig. 1.

### Main characteristics of included studies

Table 1 presents the main characteristics and genotype frequencies of the included studies. Of the 38 studies, 15 studies were carried out among Asians, 13 among Caucasians, and 10 among mixed populations. All studies included were case-control studies in design, and all patients with breast cancer fulfilled the pathological diagnosis. The number ranged from 35 to 1986 for cases, and 33 to 2414 for controls. In 21 studies, controls were normal healthy people randomly recruited from general population, and in 15 studies, controls were recruited from hospital among women with benign disease or through women going to hospital for routine physical examines, but in the two studies, we were unable to find out the source of controls. In most of the included studies, controls were matched with cases in ethnicity and age. In quality assessment, 17 studies included were categorized as “high quality,” and 21 as “moderate quality” (Table 1). In eight studies, the genotype distributions in control groups were deviated from HWE (Table 1).

**Table 1:** The main characteristics of studies included in this meta-analysis and the distribution of *MTHFR* gene *1298A>*C genotypes and alleles among cases and controls

First author	Year	Ethnicity	Source of controls	Cases			Controls			Cases		Controls		Deviation from HWE	Quality grade
				AA	AC	CC	AA	AC	CC	A	C	A	C		
Aram	2012	Caucasian	HB	35	55	20	30	75	5	125	95	135	85	Yes	+
Awwad	2015	Asian	PB	68	61	17	58	64	13	197	95	180	90	No	++
Carvalho Barbosa Rde	2012	Mixed	PB	68	80	17	72	84	9	216	114	228	102	Yes	+
Chen	2005	Mixed	PB	558	417	87	536	457	110	1533	591	1529	677	No	++
Cheng	2008	Asian	HB	207	125	19	310	207	17	539	163	827	241	Yes	+
Chou	2006	Asian	HB	104	30	8	172	95	18	238	46	439	131	No	+
Ergul	2003	Caucasian	HB	50	48	20	90	85	18	148	88	265	121	No	+
Ericson	2009	Caucasian	PB	242	242	57	487	480	105	726	356	1454	690	No	++
Forsti	2004	Caucasian	NA	94	102	27	133	127	38	290	156	393	203	No	+
Gao	2009	Asian	PB	446	165	9	425	188	11	1057	183	1038	210	No	++
He	2014	Asian	HB	138	132	40	173	155	53	408	212	501	261	No	+
Hosseini	2011	Caucasian	HB	36	96	162	60	135	105	168	420	255	345	No	+
Inoue	2008	Asian	PB	225	139	16	387	234	41	589	171	1008	316	No	++
Justenhoven	2005	Caucasian	PB	273	256	53	295	266	73	802	362	856	412	No	++
Kakkoura	2015	Mixed	PB	138	465	468	150	500	484	741	1401	800	1468	No	++
Kotsopoulos	2008	Caucasian	HB	466	390	85	398	309	73	1322	560	1105	455	No	+
Lajin	2012	Caucasian	HB	44	52	23	65	48	13	140	98	178	74	No	+
Le Marchand	2004	Mixed	PB	741	372	77	1493	801	120	1854	526	3787	1041	No	++
Lissowska	2007	Caucasian	PB	892	874	220	1086	941	251	2658	1314	3113	1443	Yes	+
Liu	2013	Asian	HB	206	176	53	214	172	49	588	282	600	270	No	+
Lopez-Cortes	2015	Mix	PB	110	3	1	191	3	1	223	5	385	5	Yes	+
Lu	2015	Asian	HB	369	172	19	352	185	23	910	210	889	231	No	+
Ma	2009	Mixed	HB	269	168	21	279	157	22	706	210	715	201	No	+
Mir	2008	Asian	NA	15	19	1	11	22	0	49	21	44	22	Yes	+
Ozen	2013	Mix	PB	17	29	5	71	35	0	63	39	177	35	Yes	+
Papandreou	2012	Caucasian	HB	129	135	36	136	116	31	393	207	388	178	No	+
Platek	2009	Mix	PB	443	402	83	842	758	181	1288	568	2442	1120	No	++
Qi	2004	Asian	PB	155	58	4	144	71	3	368	66	359	77	No	++
Sangrajrang	2010	Asian	HB	302	223	38	258	206	23	827	299	722	252	Yes	+
Sharp	2002	Caucasian	PB	27	25	3	24	25	11	79	31	73	47	No	++
Shrubsole	2004	Asian	PB	768	311	42	824	344	40	1847	395	1992	424	No	++
Stevens	2007	Mixed	PB	224	228	42	252	201	40	676	312	705	281	No	++
Vainer	2010	Caucasian	HB	398	353	80	379	330	76	1149	513	1088	482	No	+
Weiwei	2014	Asian	HB	135	129	32	151	130	25	399	193	432	180	No	+
Wu	2012	Asian	PB	37	32	6	42	28	5	106	44	112	38	No	++
Xu	2007	Mixed	PB	558	417	87	536	457	110	1533	591	1529	677	No	++
Zhang	2015	Asian	PB	98	87	31	105	84	27	283	149	294	138	No	++
Ziva Cerne	2011	Caucasian	PB	258	219	47	131	117	21	735	313	379	159	No	++

*PB* population-based study, *HB* hospital-based study, *NA* not available, *HWE* Hardy-Weinberg equilibrium

### Quantitative data analysis

#### Association between *MTHFR* gene *1298A>C* polymorphism and breast cancer susceptibility

**Fig. 2:**
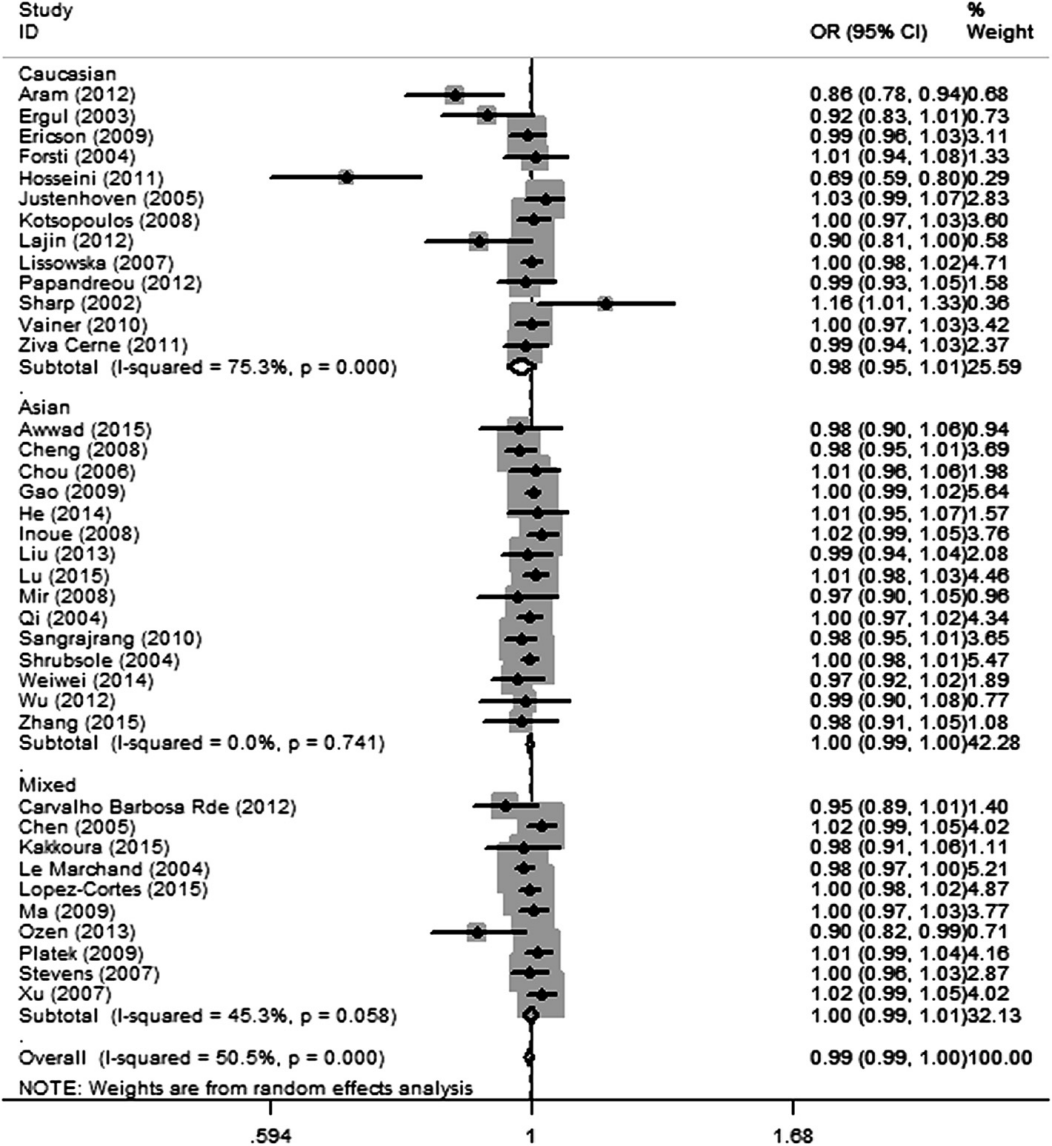
Shows the forest plot of the dominant model testing the association between *MTHFR 1298A>C* polymorphism and breast cancer risk, stratified by ethnicity

**Fig. 3:**
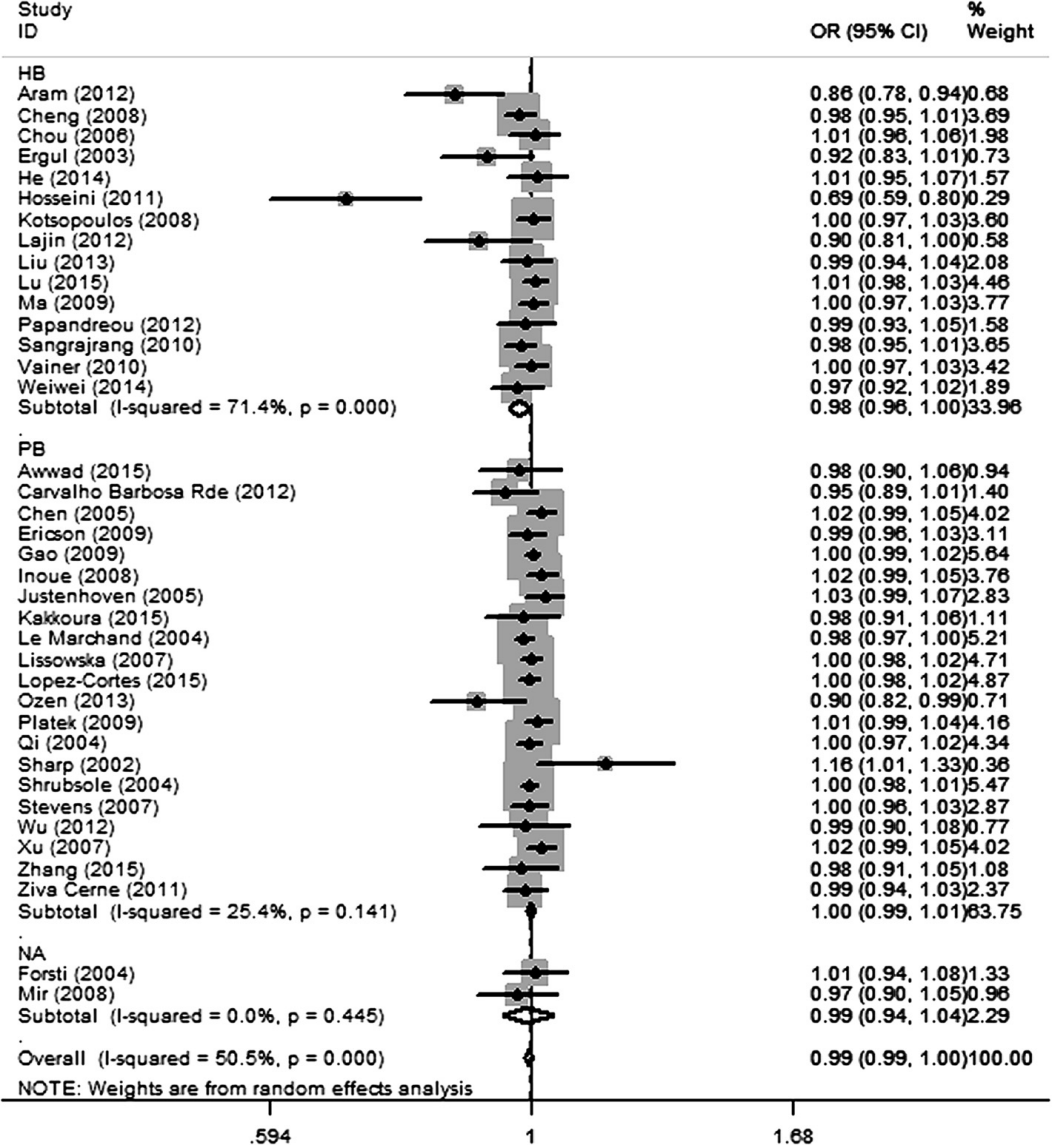
Shows the forest plot of the dominant model testing the association between *MTHFR 1298A>C* polymorphism and breast cancer risk, stratified by source of control

The results of the five genetic models testing *MTHFR* gene *1298A>C* polymorphism and breast cancer susceptibility are presented in Table 2. In the dominant model (AA + AC vs. CC), *P* value for heterogeneity was 0.000, and *I*^2^ was 50.5 %, indicating significant heterogeneity among studies. Thus, random-effect model was used. The overall effect *Z* value was 1.12 (*P* = 0.218) and OR was 0.99 (95 % CI 0.99–1.00), suggesting that no association was found in the dominant model. The Egger’s linear regression test indicated that there was some evidence of publication bias in this model (Egger, *P* = 0.01). Other four genetic models were also performed (Table 2), but no association was found. In subgroup analyses stratified by source of control, a significant decrease in breast cancer susceptibility was found in hospital-based controls in dominant model (OR = 0.98, 95 % CI 0.96–1.00, *P* = 0.037), but not in allele contrast genetic model (OR = 0.97, 95 % CI 0.94–1.00, *P* = 0.092) (Table 3). Moreover, the results showed that in subgroups of Asians and population-based studies, the heterogeneity among studies was significantly reduced. Figure 2 shows the forest plot of the dominant model testing the association between *MTHFR 1298A>C* polymorphism and breast cancer risk, stratified by ethnicity. Figure 3 shows the forest plot of the dominant model testing the association between *MTHFR 1298A>C* polymorphism and breast cancer risk, stratified by source of control.

**Table 2:** Summary of different genetic model comparison results of *MTHFR* gene *1298A>C* polymorphism

Genetic model	OR (95 % CI)	*Z*	*P* value	*I* ^2^ %	*P*_het_	Effect model	Egger’s test	
							*t* value	*P* value
AA + AC vs. CC	0.99 (0.99–1.00)	1.23	0.218	50.5	0.000	R	−2.72	0.010
AA vs. AC + CC	1.00 (0.97–1.02)	0.15	0.880	35.9	0.016	R	−1.45	0.155
AA vs. CC	0.99 (0.98–1.01)	0.86	0.390	43.8	0.002	R	−2.75	0.014
AC vs. CC	0.99 (0.97–1.00)	1.48	0.138	41.2	0.005	R	−2.55	0.015
A vs. C	0.99 (0.98–1.01)	0.92	0.360	55.5	0.000	R	−2.27	0.029

*OR* odds ratio, *CI* confidence interval, *R* random-effect model, *P*_het_ *P* value for heterogeneity

*P* < 0.05 stands for statistical significance

**Table 3:** Results of subgroup analyses of *MTHFR* gene *1298A>C* polymorphism

Stratified by	Comparison	Number of datasets	Dominant genetic model		Allele contrast	
			OR (95 % CI)	*P* value	OR (95 % CI)	*P* value
Ethnicity	Asian	15	1.00 (0.99–1.00)	0.506	1.01 (0.99–1.02)	0.249
	Caucasian	13	0.98 (0.95–1.01)	0.129	0.97 (0.93–1.00)	0.059
	Mixed	10	0.99 (0.99–1.00)	0.852	0.99 (0.98–1.01)	0.660
Source of control	PB	21	1.00 (0.99–1.01)	0.931	1.00 (0.99–1.02)	0.830
	HB	15	0.98 (0.96–1.00)	0.037	0.97 (0.94–1.00)	0.092
	NA	2	0.99 (0.94–1.04)	0.793	0.99 (0.91–1.08)	0.892
Deviation from HWE	Yes	8	0.98 (0.95–1.00)	0.019	0.98 (0.95–1.01)	0.102
	No	30	1.00 (0.99–1.01)	0.909	1.00 (0.98–1.01)	0.801

*PB* population-based study, *HB* hospital-based study, *NA* not available

#### Sensitivity analysis and publication bias

Sensitivity analyses were conducted by omitting each dataset sequentially, and the result did not change under any genetic model. Sensitivity analysis suggested that for all of the five genetic comparisons of *MTHFR* gene *1298A>C* polymorphism and breast cancer susceptibility, the results were statistically robust.

**Fig. 4:**
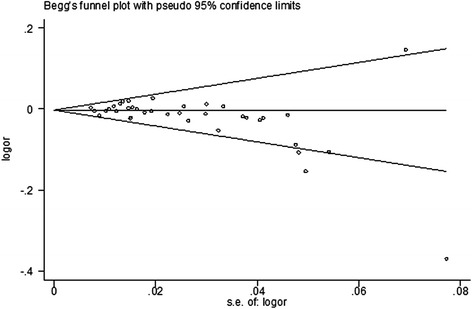
Begg’s funnel plot under dominant model of *MTHFR 1298A>C* polymorphism

Visual inspection of Begg’s funnel plots identified the substantial asymmetry for dominant model, the allele contrast genetic model, the heterozygote comparison, and the homozygote comparison. The Egger’s linear regression test also indicated the similar results (*P* < 0.05 for all models tested except the recessive genetic model) (Table 2). Figure 4 shows the Begg’s funnel plot under dominant model of *MTHFR 1298A>C* polymorphism.

## Discussion

*MTHFR* is an essential gene in the one-carbon metabolism pathway. During the past few years, many meta-analyses assessing the association between *MTHFR* gene polymorphism and cancer risks have been published, including liver cancer, ovary cancer, lung cancer, gastric cancer, pancreatic cancer, cervical cancer, and esophageal cancer [56–60]. Genetic variation in enzymes and proteins involved in folate metabolism is also a rational candidate for studying the genetic of breast cancer. Therefore, the interest in *MTHFR* gene *1298A>C* polymorphism and breast cancer susceptibility has existed for a long time. In 2002, Sharp et al. for the first time published a case-control study estimating the association between *MTHFR* gene *1298A>C* polymorphism and breast cancer risk. Their result suggested that risk was significantly lower for the *1298CC* genotype compared to *AA* genotype (OR = 0.24, 95 % CI 0.06–0.97) [49]. However, after that, a number of subsequent studies were conducted and their results were inconsistent, with some studies showed significant associations while others were not. The inconsistency may be caused by several reasons. First of all, although in vitro, the variant genotype is associated with a substantial decrease in enzymatic activity [11], this functional polymorphism may be an important but not the exclusive influencing factor in etiology and pathogenesis of breast cancer. Special lifestyle and environmental factors, such as tea drinking [61], dietary intake of folate, vitamin B_6_ and B_12_ [62], physical activities [63], long-term oral contraceptive use [64], and hormone replacement therapy use [65], are possibly confounding factors taking part in the disease etiology. Moreover, differences in patient choosing criteria, ethnicity, sample size, and sources of control could contribute to inconsistency. Hence, it is necessary to conduct a meta-analysis providing quantitative approach for pooling the results of all studies with the same purpose and explaining the overall estimation as well as the diversity.

Our study has important strengths. All original studies used a case-control study design, which is a useful tool to identify gene and disease associations. However, individual genotype case-control studies could not be based on a large number of subjects or contain patients in different ethnicities, and thus has insufficient statistical power. Our meta-analysis based on case-control studies involving 40,985 subjects brings to light that there is no significant association between *MTHFR* gene *1298A>C* polymorphism and breast cancer susceptibility for overall population, with ORs from 0.99 to 1.00 and narrow 95 % CIs for all of the five genetic models. Moreover, in our study, no association was found in different ethnicities or in population-based studies, which thereby strengthened this association. As shown in our meta-analysis, studies with hospital-based design or controls deviated from HWE had a weak, but statistical significant decreased association with breast cancer in dominant model. However, in these two kinds of studies, the controls may not represent the whole population and thereby, the results from them should be interpreted with caution. Overall, our meta-analysis based on 38 case-control studies provided reliable and comprehensive estimations. The association in the five genetic models sustained unchanged in the sensitivity analysis, which further confirmed the results of main analysis.

It is also important to mention the heterogeneity existed in this study. For all genetic models in the main analysis, *P* value for heterogeneity was less than 0.05, indicating significant heterogeneity among the included studies. Finding the potential sources of heterogeneity is an important part of meta-analysis, which can greatly influence the results of the research. To detect the possible source of heterogeneity in our meta-analysis, we performed the subgroup analysis stratified by ethnicity, source of control, and deviation from HWE. When stratified by ethnicity and source of control, the heterogeneity was significantly decreased in Asian and population-based subgroups. Therefore, the different ethnicity and source of control may contribute to the overall heterogeneity. However, heterogeneity still existed in Caucasian, mixed ethnicity, and hospital-based control subgroups, suggesting that ethnicity and source of control did not fully explain the heterogeneity among studies. Further studies may try to explore in interactions between different factors and to minimize the heterogeneity in subgroups.

Several previous meta-analyses have been published to analyze the association between *MTHFR* gene polymorphisms and breast cancer susceptibility, and the majority of them concerned on *677C>T* polymorphism [66–69]. Two studies published in 2014 have detected the *1298A>C* polymorphism [17, 70]. The main result of our study was consistent with the previous meta-analyses. Comparing with these two studies, our study has some important improvements. In 2014–2015, some new studies were published and they were included in our meta-analysis. Through strict methodological process, we provided a more comprehensive view of included studies. The abovementioned meta-analyses only stratified by ethnicity to test if there existed differences in variant ethnicities. In present study, we also conducted subgroup study stratified by source of control and deviation from HWE in control group, to analyze if there were differences between subgroups.

We should also pay attention to the several limitations in our study, which may affect the result. Firstly, we only included published studies meeting our inclusion criteria from two databases, similar studies in other databases and unpublished researches may have been missed, and this is also the main reason for the publication bias we found in four of the five genetic models. Secondly, the control groups in some of the included studies were deviated from HWE, which may fail to represent the whole population and have some effects on the overall estimation. Thirdly, although the results from subgroup and sensitivity analyses were quite similar to the main analysis, significant heterogeneity was detected in all five genetic models of *MTHFR* gene *1298A>C* polymorphism and breast cancer susceptibility. Different characteristics in study population and study design may contribute to the heterogeneity. Considering that meta-analysis is a kind of retrospective research and may easily be affected by methodological deficiencies of the included studies, we developed a detailed protocol before conducting this analysis, to ensure the quality of our research.

## Conclusions

From the combination results of currently included studies, our meta-analysis suggested that there is no significant association between *MTHFR* gene *1298A>C* polymorphism and breast cancer susceptibility for overall population.

## Abbreviations

MTHFR: Methylenetetrahydrofolate reductase
OR: Odds ratio
SNPs: Single nucleotide polymorphisms
HWE: Hardy-Weinberg equilibrium
